# Serum-Derived Exosomal miR-140-5p as a Promising Biomarker for Differential Diagnosis of Anti-NMDAR Encephalitis With Viral Encephalitis

**DOI:** 10.3389/fimmu.2022.840003

**Published:** 2022-02-22

**Authors:** Xiaofeng Liu, Kengna Fan, Qingwen Lin, Minjie Tang, Qi Wang, Er Huang, Weiqing Zhang, Tianbin Chen, Qishui Ou

**Affiliations:** ^1^Department of Laboratory Medicine, the First Affiliated Hospital of Fujian Medical University, Fuzhou, China; ^2^Fujian Key Laboratory of Laboratory Medicine, The First Affiliated Hospital, Fujian Medical University, Fuzhou, China; ^3^Gene Diagnosis Research Center, the First Affiliated Hospital of Fujian Medical University, Fuzhou, China

**Keywords:** anti-NMDAR encephalitis, viral encephalitis, exosome, miRNAs, biomarker

## Abstract

**Background:**

Anti-*N*-methyl-D-aspartate receptor (anti-NMDAR) encephalitis is the most common type of autoimmune encephalitis. Early recognition and treatment, especially distinguishing from viral encephalitis (VE) in the early stages, are crucial for the outcomes of patients with anti-NMDAR encephalitis. Compared with plasma microRNAs (miRNAs), exosomal miRNAs are more abundant and not easy to degrade. Moreover, exosomes can pass through the blood–brain barrier. This study aimed to explore the clinical value of serum exosomal miRNAs in the differential diagnosis of anti-NMDAR encephalitis with VE.

**Method:**

Serum samples from a total of 30 patients with anti-NMDAR encephalitis, 30 patients with VE, and 30 cases of control patients hospitalized in the same period were collected. Firstly, the serum exosomes were isolated and identified by transmission electron microscope (TEM), nanoparticle-tracking analyzer (NTA), and Western blot (WB). The expression levels of let-7b and miR-140-5p from serum exosomes were detected by real-time quantitative PCR (qPCR). At the same time, we also detected complement 3 (C3), complement 4 (C4), and high sensitivity CRP (hs-CRP) expression levels in three groups. Finally, we analyzed the difference and diagnostic value of the test results.

**Results:**

Isolated particles showed identical characteristics to the exosomes through TEM, NTA, and WB analyses. Compared with the VE group and control group, the expression of miR-140-5p was significantly upregulated in serum exosomes of the NMDAR group. In contrast, the serum C3 in the NMDAR group was significantly lower than the other two groups. ROC curve analysis showed the area under the curve (AUC) of serum exosomal miR-140-5p and serum C3 was 0.748 (76.67% sensitivity and 73.33% specificity) and 0.724 (76.67% sensitivity and 60% specificity) to distinguish anti-NMDAR encephalitis from VE, respectively. The AUC of serum exosomal miR-140-5p combined with serum C3 was 0.811, the sensitivity was 70.00%, and the specificity was 86.67%.

**Conclusion:**

Serum exosomal miR-140-5p combined with serum C3 would be a promising marker in the differential diagnosis of anti-NMDAR encephalitis with VE.

## Introduction

Autoimmune encephalitis (AE) is an inflammatory disease of the central nervous system in which antibodies mediate neuronal damage and cause structural dysfunction (1). Anti-*N*-methyl-D-aspartate receptor encephalitis (anti-NMDAR encephalitis) is the most common subtype of AE (1), which was characterized by a series of complex neuropsychiatric symptoms and expresses antibodies against subunits of NMDAR GluN1 in CSF (2). The clinical manifestations of anti-NMDAR encephalitis are diverse, and the electroencephalogram (EEG) and imaging examinations lack specificity. The diagnosis is mainly reliant on the positive result of CSF or serum antibodies. However, antibody examinations were not popularized and some hospitals need to send them to a third-party institution for testing, which takes about 3–5 days, delaying diagnosis and worsening patients’ economic and emotional burden (3). In addition, the clinical symptoms, cerebrospinal fluid (CSF) tests, and brain MRI are similar between the disease of AE and VE. The detection rate of VE pathogens is low; therefore, VE is mainly diagnosed on the basis of epidemiology, clinical manifestations, CSF detection, imaging, EEG and of excluding other diseases. In conclusion, it is difficult to diagnose patients with anti-NMDAR encephalitis in the early stage.

Exosomes are small vesicles of about 30–150 nm in body fluid, mainly formed by invagination of lysosomal microparticles in the cell, rich in RNA and protein participating in intercellular communication (4). MicroRNA (miRNA) is a highly conservative short-chain noncoding RNA that plays a vital role in the central nervous system. It has been studied in various central nervous system diseases such as ischemic stroke, Alzheimer’s disease, and tuberculous meningitis (5–7). It has been confirmed that blood miRNAs are mainly derived from exosomes. Compared with plasma miRNAs, exosomal miRNAs are more abundant and stable (8). In addition, exosomes can pass through the blood–brain barrier, so the serum exosomal miRNAs can also reflect the characteristics of miRNAs in the central nervous system (9), which is a promising marker for neurological diseases.

Recent studies have shown that let-7b and miR-140-5p play a certain role in the central nervous system and participate in the processes of apoptosis, tumorigenesis, and inflammatory progression of nerve cells (10–12). However, these above miRNA-derived serum exosomes have not yet been reported in the anti-NMDAR encephalitis or VE. This study aimed to isolate serum exosomes and explore the value of serum exosomal let-7b and miR-140-5p in the anti-NMDAR encephalitis, especially the value of differential diagnosis between anti-NMDAR encephalitis and VE.

## Materials and Methods

### Study Subjects

From October 2018 to November 2020, we collected data from 30 cases of anti-NMDAR encephalitis, 30 cases of VE, and 30 cases of noninflammatory neurologic disorders as control patients with matching age and sex in the first affiliated Hospital of Fujian Medical University (including mental disorder, cerebrovascular disease, neurodegenerative diseases, respiratory tract infection, syphilis not involving the central nervous system, ataxia, dyskinesia, peripheral neuropathy, and so on).

We collected 5 ml serum stored at − 80°C for further analysis and clinical data of the above three groups of patients. This study has been approved by the Ethics Committee of the First Affiliated Hospital of Fujian Medical University (No, MRCTA, ECFAH of FMU, 2020[324]). Each participant signed written informed consent before recruitment.

### Exosome Isolation and Identification

The Serum Exosome Isolation Kit (#UR52136, Umibio Co., Ltd., Shanghai, China) was used to isolate exosomes according to the manufacturer’s instructions. Finally, 200-μl suspension solution was obtained and stored at − 80°C for further analysis. Transmission electron microscopy (TEM), nanoparticle-tracking analysis (NTA), and Western blot (WB) analysis including CD63 and TSG101 were used to confirm the characters of exosomes.

### Detection of Serum Exosomal miRNA

Firstly, according to the experimental procedure by the miRNA Purification Kit (CW0627, CWBIO, Taizhou, China), 200 μl exosomes eventually obtained 30 μl total miRNA. Secondly, miRNA 1st Strand cDNA Synthesis Kit (MR101-01, Vazyme, Nanjing, China) was used to reverse transcripted cDNAs with a stem-loop RT primer. All primers were synthesized by Sangon Biotech Co., Ltd. (Shanghai, China), and primer sequence information is shown in **Table 1**. Finally, exosomal miRNAs (let-7b and miR-140-5p) were measured *via* real-time PCR using miRNA Universal SYBR qPCR Master Mix (MQ101-01, Vazyme, Nanjing, China). U6 was used as the internal reference, and cel-miR-39 was used as the external reference. PCR reaction was performed in triplicate and analyzed using a QuantStudio Dx qPCR system (Applied Biosystems, Foster City, CA, USA).

**Table 1:** Gene primers.

Name	RT primers (5′-3′)	Forward primers (5′-3′)
U6	AACGCTTCACGAATTTGCGT	CTCGCTTCGGCAGCACA
let-7b	GTCGTATCCAGTGCAGGGTCCGAGGTATTCGCACTGGATACGACAACCAC	GCGCGTGAGGTAGTAGGTTGT
miR-140-5p	GTCGTATCCAGTGCAGGGTCCGAGGTATTCGCACTGGATACGACCTACCA	CGCGCAGTGGTTTTACCCTA
Universal reverse primers	AGTGCAGGGTCCGAGGTATT (5′-3′)	

### Detection of Serum Complement and hs-CRP

The levels of serum complement 3 (C3) and complement 4 (C4) were measured on IMMAGE 800 Immunochemistry System (Beckman Coulter, Brea, CA, USA). High-sensitivity CRP (hs-CRP) level was measured on ADVIA 2400 Chemistry System (Siemens Healthineers, Erlangen, Germany).

### Statistics

Normally distributed data was expressed as the mean ± standard deviation (mean ± SD), and Student’s t-test was used to compare the two groups. One-way analysis of variance was used for the comparison among the multiple groups. In nonnormally distributed data, the median and interquartile range (m, Q) was used to show the characteristic of the data, and the Mann–Whitney *U* test was used to compare the outcomes. The Kruskal–Wallis *H* test was used for the comparison among the multiple groups. The relative expression values were normalized to U6 and miR-39 and calculated by the 2^−ΔΔCt^ method. The receiver operating characteristic (ROC) curve was performed by the MedCalc system. *p* < 0.05 was statistically significant.

## Results

### Clinical Characteristics of Subjects

In our study, we enrolled 30 patients with NMDAR, 30 patients with VE, and 30 age- and sex-matched control group (CG), respectively. As shown in **Table 2**, age and sex were not significantly different among the three groups. Seizure (63.3%) was the most common clinical presentation in the NMDAR group, while fever was the most common clinical presentation in the VE group (66.7%). White blood cell count in CSF of patients was notably increased in the NMDAR group and VE group compared with the CG group. All patients in the NMDAR group had positive result in the test of serum or CSF antibody. Only two anti-NMDAR patients had teratoma comorbidity, while one VE patient had hypophysoma. Some patients in each group had been associated with infectious diseases.

**Table 2:** Clinical characteristics of patients in three groups.

	NMDAR group	VE group	Control group	*p-*value
Total number (*n*)	30	30	30	–
Age (years, mean ± SD)	24.3 ± 13.3	29.2 ± 17.3	30.6 ± 20.5	0.341
Sex [M (*n*, %)]	15 (50.0%)	14 (46.7%)	13 (43.3%)	0.876
Clinic symptoms (*n*, %)				
Fever	8 (26.7%)	20 (66.7%)	8 (26.7%)	0.001
Psychiatric symptom	15 (50%)	6 (20%)	3 (10%)	0.001
Abnormal movements	15 (50.0%)	5 (16.7%)	4 (13.3%)	0.002
Disorders of memory	8 (26.7%)	1 (3.3%)	1 (3.3%)	0.005
Seizure	19 (63.3%)	7 (23.3%)	3 (10%)	<0.001
Disorders of sleep (*n*, %)	5 (16.7%)	1 (3.3%)	1 (3.3%)	0.099
CSF routine [M (Q1, Q3)]				
WBC (×10^6^/L)	13 (3.8, 30.3)	23 (7.8, 63.5)	2 (1.0, 3.3)	<0.001
GLU (mmol/L)	2.8 (2.6, 3.4)	3.08 (2.8, 3.4)	2.89 (2.6, 3.4)	0.239
Cl (mmol/L)	119.0 (117.0, 121.0)	119.5 (116.8, 123.0)	120.0 (119.0, 121.0)	0.279
Serum/CSF anti-NMDAR antibody positive (*n*, %)	30 (100%)	0	0	–
Tumor comorbidity (*n*, %)	2 (6.7%)	1 (3.3%)	0	0.355
Infectious disease (*n*, %)	9 (30.0%)	14 (46.7%)	11 (36.7%)	0.407

NMDAR, anti-N-methyl-d-aspartate receptor encephalitis; VE, viral encephalitis; M, male.

### Characterization of the Isolated Serum Exosomes

**Figure 1:**
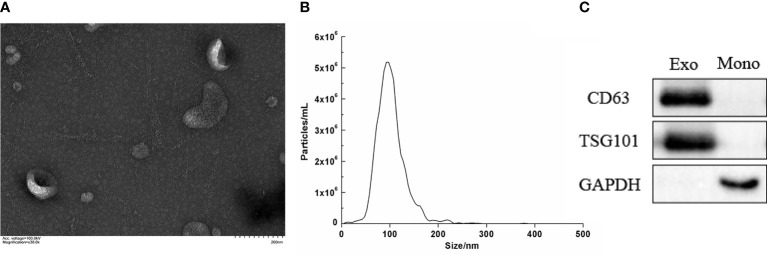
Characterization of the isolated serum exosomes. **(A)** Morphology of serum exosome by electron transmission microscope (×30.0k). **(B)** Concentration and size distribution of serum exocrine. **(C)** The expression of the surface-specific protein of exosome by Western blot. (Exo, exosomes; Mono, mononuclear cell; CD63 and TSG101 were exosome markers).

Serum-derived vesicles showed a circular, round, and cup morphology with a diameter of about 30–150 nm by TEM. The vesicles were coated with a phospholipid bimolecular layer and contained a low electron density substance (**Figure 1A**). Furthermore, the NTA measurements revealed that our serum particles’ average size and main peak were about 100 nm, and the concentration was 5.9 × 10^9^/mL (**Figure 1B**). Moreover, the exosome markers, including CD63 and TSG101, were positively expressed in the isolated particles, with low expression of GAPDH. The expression of the above protein in the mononuclear cell was opposite, where the high expression of GAPDH and no expression of CD63 and TSG101 are shown (**Figure 1C**). The characteristics described above are consistent with exosomes (4), indicating that we have successfully obtained exosomes from serum.

### The Expression Levels of let-7b and miR-140-5p in Serum Exosomes

**Figure 2:**
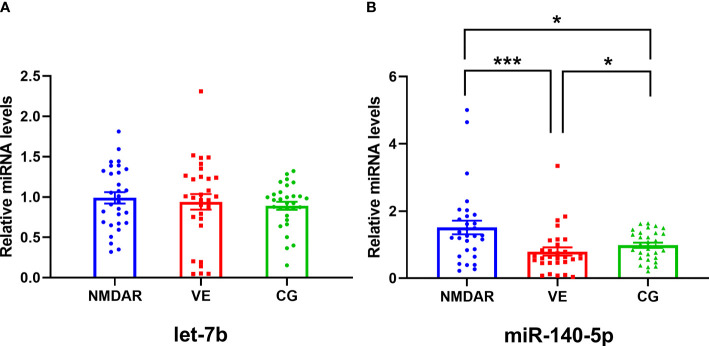
The expression levels of let-7b and miR-140-5p in serum exosomes in three groups. **(A)** The expression level of let-7b among the NMDAR, VE, and CG groups. **(B)** The expression level of miR-140-5p among the NMDAR, VE, and CG groups. (^***^p < 0.001; ^*^p < 0.05; NMDAR, anti-*N*-methyl-d-aspartate receptor encephalitis; VE, viral encephalitis; CG, control group).

Expression levels of let-7b in the NMDAR, VE, and CG groups were analyzed. As shown in **Figure 2**, the relative expression level of let-7b in the serum exosomes of the NMDAR group was the highest, but there was no significant difference in the three groups (all *p* > 0.05). Meanwhile, we analyzed the expression level of miR-140-5p in the three groups. The miR-140-5p expression in the NMDAR group was significantly higher than that in the VE group (*p* < 0.001) and the CG group (*p* < 0.05). In addition, the expression level of miR-140-5p in the CG group was higher than that of the VE group, and the difference was statistically significant (all *p* < 0.05).

### The Expression Levels of Serum C3, C4, and hs-CRP

**Figure 3:**
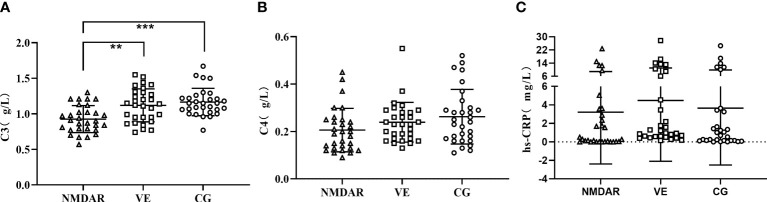
The expression levels of serum C3, C4, and hs-CRP. **(A)** The expression level of C3 among three groups. **(B)** The expression level of C4 among three groups. **(C)** The expression level of hs-CRP among three groups. (^***^p < 0.001; ^**^p < 0.01; NMDAR, anti-*N*-methyl-d-aspartate receptor encephalitis; VE, viral encephalitis; CG, control group; C3, complement 3; C4, complement 4; hs-CRP, high sensitivity CRP).

The expression of C3, C4, CH50, and CRP in anti-NMDAR encephalitis has been reported as abnormal and correlated with disease severity (13). Therefore, we detected serum C3, C4, and hs-CRP in the three groups. The expression of C3 in the NMDAR group was the lowest and significantly lower than that of the VE group and CG group (NMDAR group: 0.926 ± 0.191 g/L; VE group: 1.119 ± 0.232 g/L; CG group: 1.166 ± 0.193 g/L) (*p* < 0.05) (**Figure 3**). The expression of C4 in the NMDAR group was slightly lower than the other two groups, but there was no significant difference (NMDAR group: 0.206 ± 0.0.092 g/L; VE group: 0.234 ± 0.084 g/L; CG group: 0.262 ± 0.115 g/L) (*p* >0.05) (**Figure 3**). There was no significant difference in the expression of hs-CRP in the three groups (NMDAR group: 3.212 ± 5.601 mg/L; VE group: 4.484 ± 6.585 mg/L; CG group: 3.643 ± 6.151 mg/L) (all *p* > 0.05, **Figure 3**).

### Differential Diagnostic Value of Serum Exosomal miR-140-5p and Serum C3

**Figure 4:**
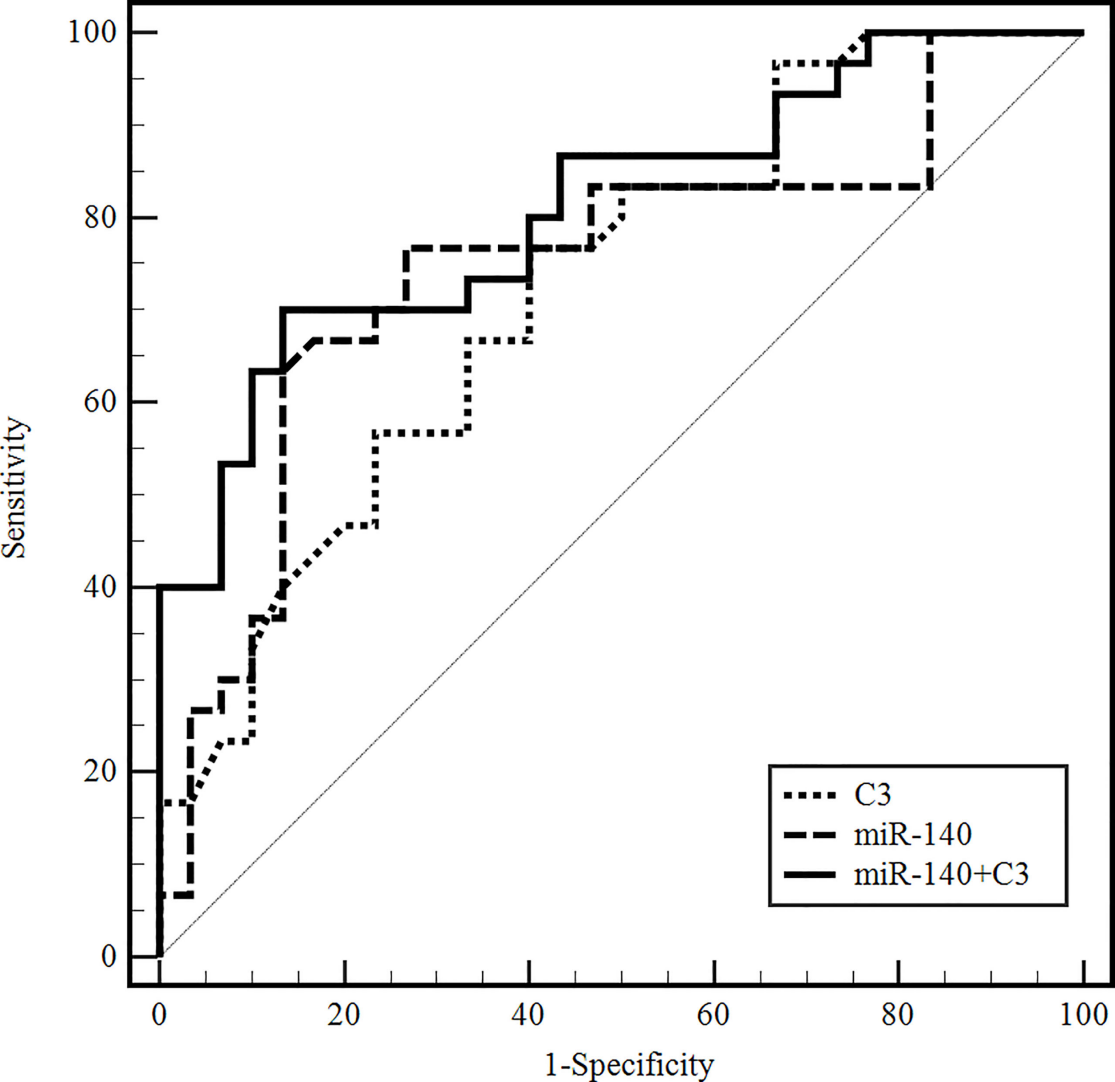
ROC curves to validate the differential diagnostic value of exosomal miR-140-5p, serum C3, and their combinations for anti-NMDAR encephalitis. (C3, complement 3; C4, complement 4; miR-140, microRNA-140-5p).

We later used the ROC curve to evaluate the differential diagnosis value of miR-140-5p and serum C3 in the NMDAR group against the VE group. The area under the curve (AUC) of serum exosomal miR-140-5p was 0.748 (95% CI: 0.620–0.851), and the sensitivity was 76.67% at the specificity of 73.33%. The AUC of serum C3 alone was 0.724 (95% CI: 0.594–0.832), and the sensitivity was 76.67% at the specificity of 60%. The AUC of serum exosomal miR-140-5p combined with serum C3 was 0.811 (95% CI: 0.661–0.881), and the sensitivity was 70.00% at the specificity of 86.67% (**Figure 4**).

## Discussion

Since Dalmau et al. firstly reported anti-NMDAR encephalitis in 2007 (14), the anti-NMDAR encephalitis has become the most common type of AE in clinical practice. However, the pathogenesis of anti-NMDAR encephalitis remains unclear. There are some hypotheses, such as tumor tissue antigen-activated autoimmune reaction (15), antigen exposure induced after viral infection (16, 17), and immune clearance disorder (18). Moreover, the disease progression of anti-NMDAR encephalitis is fast and serious, and early detection can significantly improve the disease and prognosis. However, the clinical manifestations of anti-NMDAR encephalitis are diverse, and laboratory tests, brain MRI, or EEG are nonspecific, similar to that of VE. Therefore, it is still a huge challenge to identify the two diseases before autoantibody or pathogen detection results are confirmed (16, 19). Therefore, it is significant to find biomarkers that can be used for early identification of anti-NMDAR encephalitis, especially to distinguish from VE.

To date, few studies have reported the serum exosomal miRNAs in patients with anti-NMDAR encephalitis. Recent studies have shown that exosomal miRNAs are more abundant than plasma miRNAs and are less susceptible to degradation (8). Moreover, exosomes can cross the blood–brain barrier into the peripheral blood, allowing serum exosomal miRNAs to represent central nervous system miRNA properties without invasive examination (9). In this study, we focused on the value of serum exosomal miRNAs as differential diagnostic biomarkers of anti-NMDAR encephalitis. Our results showed that the serum exosomal miR-140-5p expression in the NMDAR group was significantly higher than that in the VE and CG groups. The ROC analysis showed that serum exosomal miR-140-5p combined with serum C3 may serve as a promising biomarker for the differential diagnostic of anti-NMDAR encephalitis.

After separating the serum exosomes, we first used TEM to observe its characteristic, which was about 30-150 nm diameter, with the shape of circular, round and cup, and wrapped with phospholipid double molecular layer and contained low electron density substances internal. Furthermore, NTA showed the serum exosomes’ concentration was 5.9 × 10^9^/mL and the average diameter was 100 nm. Finally, WB analysis showed a high expression of CD63 and TSG101 in serum exosomal protein. The above results were consistent with the characteristics of the exosomes (4).

Let-7b is a member of the let-7 family and is involved in the development of multiple diseases, such as the let-7b/TLR4 pathway. It can promote macrophage differentiation and chronic inflammation regression and participate in the inflammatory process (20); Zhang et al. (21) showed that the expression of plasma let-7b in the anti-NMDAR encephalitis treatment group was significantly downregulated compared with the control group. However, it is not clear whether the expression of let-7b in the serum exosomes of AE patients was changed. In this study, we found that the expression of let-7b in serum exosomes was not significantly different between the NMDAR, VE, and CG groups. Considering that most of the serum samples in the NMDAR group were in the pretreatment stage and not yet treated with medication, there was no difference in the serum exosomal let-7b.

It has been reported that miR-140-5p was associated with many diseases such as cardiocerebral vascular diseases, tumors, autoimmune diseases, etc. Guan et al. (22) found that the expression of miR-140-5p was negatively correlated with multiple sclerosis, and miR-140-5p inhibited CD4+ T-cell differentiation and STAT1 activation, thus slowing the progression of multiple sclerosis. Zhu et al. (23) reported that miR-140-5p was involved in the pathogenesis of autoimmune encephalomyelitis by regulating Th1 differentiation through DNA methylation and mitochondrial pathway. It has been reported that miR-140-5p was involved in the progression of inflammatory and autoimmune diseases, but no scholars have reported the relationship between miR-140-5p and anti-NMDAR encephalitis. This study showed that the expression of serum exosomal miR-140-5p was significantly higher in the NMDAR group than in the VE and CG groups. A recent study had shown that the PI3K-AKT pathway participated in the activation and proliferation of B lymphocytes of systemic lupus erythematosus; PTEN inhibited the occurrence of this pathway (24). Yin et al. (25) showed that miR-140 inhibited PTEN expression. Therefore, we speculated that miR-140-5p might be involved in the activation and proliferation of B cells *via* the PI3K-AKT pathway by inhibiting the expression of PTEN in anti-NMDAR encephalitis, and then participated in the production of autoantibodies. We then used the ROC curve to evaluate the diagnostic efficacy of miR-140-5p between the NMDAR and VE groups, which found that the AUC, sensitivity, and specificity were respectively 0.748, 76.67%, and 73.33%, whereby the diagnostic efficacy needs to be improved.

Based on the study of miR-140-5p involvement in the immune response described above, C3, C4, and CRP expression in anti-NMDAR encephalitis were abnormal and correlated with disease severity (13); we speculated whether miR-140-5p is related to the above complement system. Therefore, we examined the levels of C3, C4, and hs-CRP among the three groups. The results showed that the expression of C3 in the NMDAR group was the lowest and significant, while the expression of C4 and hs-CRP was not significantly different among the three groups. Subsequently, we found no correlation between miR-140-5p and C3, C4, and hs-CRP.

Next, we further evaluated whether the combination of serum exosomal miR-140-5p and serum C3 could improve the differential diagnosis efficacy against anti-NMDAR encephalitis and VE using the ROC curve analysis. The AUC value of the serum exosomal miR-140-5p combined with serum C3 was 0.811, with 70.00% sensitivity and 86.67% specificity, which was higher than that of serum exosomal miR-140-5p or serum C3, alone.

To our knowledge, this is the first study to explore the possible value of serum exosomal miRNAs as differential diagnostic markers of anti-NMDAR encephalitis. We found a significant association of serum exosomal miRNAs with anti-NMDAR encephalitis. In addition, serving as a biomarker, serum exosomal miRNAs is convenient without invasive examination. At the same time, there are some limitations in this study. The sample size analyzed for each group is relatively small. Additionally, in the present study, we focused on the study of exosomal miRNAs from serum and have not yet been compared with exosomal miRNAs from CSF in patients with anti-NMDAR encephalitis. Future study will be needed to explore the value of CSF exosomal miRNAs in patients with anti-NMDAR encephalitis.

In conclusion, our results suggested that serum exosomal miR-140-5p expression was significantly elevated in patients with anti-NMDAR encephalitis, and serum exosomal miR-140-5p combined with serum C3 would be a promising biomarker for the differential diagnosis of anti-NMDAR encephalitis and VE.

## Data Availability Statement

The original contributions presented in the study are included in the article/supplementary material. Further inquiries can be directed to the corresponding author.

## Ethics Statement

The studies involving human participants were reviewed and approved by The Ethics Committee of the First Affiliated Hospital of Fujian Medical University. The patients/participants provided their written informed consent to participate in this study.

## Author Contributions

XFL and QSO put forward the concept and designed the study. KNF, QWL, and QW performed clinical data and serum sample collection. XFL, KNF, MJT, and QW carried out experiments. EH, WQZ, TBC, and QSO analyzed the data. XFL and KNF wrote the manuscript. XFL and QSO reviewed the manuscript and finalized the paper. All authors read and approved the final manuscript.

## Funding

This work was supported in part by grants from the National Natural Science Foundation of China (81802088), the Joint Funds for the Innovation of Science and Technology, Fujian Province (2018Y9080), and the Startup Fund for scientific research, Fujian Medical University (2019QH1094).

## Conflict of Interest

The authors declare that the research was conducted in the absence of any commercial or financial relationships that could be construed as a potential conflict of interest.

## Publisher’s Note

All claims expressed in this article are solely those of the authors and do not necessarily represent those of their affiliated organizations, or those of the publisher, the editors and the reviewers. Any product that may be evaluated in this article, or claim that may be made by its manufacturer, is not guaranteed or endorsed by the publisher.
